# Application of principles of cognitive psychology in teaching: Perspectives from undergraduate medical and dental students

**DOI:** 10.1371/journal.pone.0317792

**Published:** 2025-02-06

**Authors:** Ambreen Surti, Shaur Sarfaraz, Rabiya Ali, Mukhtiar Baig, Rehana Rehman

**Affiliations:** 1 Department of Biological & Biomedical Sciences, Aga Khan University, Karachi, Pakistan; 2 Department of Medical Education, Altamash Institute of Dental Medicine, Karachi, Pakistan; 3 Department of Physiology, Karachi Institute of Medical Sciences (KIMS) NUMS, CMH, Karachi, Pakistan; 4 Department of Clinical Biochemistry, Faculty of Medicine, Rabigh, King Abdulaziz University, Jeddah, Kingdom of Saudi Arabia; Ajman University, UNITED ARAB EMIRATES

## Abstract

**Introduction:**

Principles of cognitive psychology (CP) aim to shed light on the fundamentals of perception, attention, and knowledge extraction used for critical thinking, learning, and recollection of information. These principles were incorporated to educate undergraduate medical and dental students, and the study aims to assess the perspectives of medical and dental students regarding applying these principles.

**Methods:**

The descriptive cross-sectional study was carried out among 555 Bachelor of Dental Surgery (BDS) and Bachelor of Medicine, Bachelor of Surgery (MBBS) students using a validated questionnaire with purposive sampling. Data was analyzed on SPSS version 21.

**Results:**

The study population comprised 555 undergraduate medical and dental students, with a mean age of 20.55 ± 1.86 years. Of these, 63.4% were pursuing MBBS, and 36.6% were BDS students. The sample included 320 (57.65%) female and 235 (42.35%) male students. MBBS and BDS students exhibited high confidence levels in most aspects of CP principles required for interactive learning. However, they expressed lower confidence in facilitator-student interaction, receiving feedback within large classes, and experiencing online teaching elements. A significant difference was observed between the two groups. In five of six CP attributes, MBBS students demonstrated significantly higher perceptions than BDS students: overcoming cognitive and emotional challenges, recognizing and overcoming ineffective learning strategies, paying attention in class, and integrating knowledge (p < 0.05).

**Conclusion:**

The current study reveals that MBBS students perceived the application of CP principles more positively than BDS students in key interactive learning areas. Furthermore, the integration of CP principles enhanced session interactivity, student engagement, attention, and retention. To optimize learning outcomes, institutions should consider adopting blended learning strategies, curricular innovations, and active learning methodologies (such as case-based, team-based, and problem-solving approaches) aligned with CP principles. Future longitudinal research could provide deeper insights into the long-term impact of CP principles on student learning and perception.

## Introduction

Educators who thoughtfully and carefully apply the principles of cognitive psychology (CP) in developing their lesson plans to teach undergraduate medical and dental students can significantly impact achieving outcomes and developing competent clinical practitioners [1].

The main objective of teaching is to provide an environment in which students are able to absorb the educational material of the respective program. Teachers need to be trained and required to incorporate cognitive ideas into their teaching methods to provide students with opportunities to encode and retrieve knowledge when required [2]_._ For this purpose, CP aims to shed light on how people solve problems through perception, attention, knowledge extraction, critical thinking, learning, and recollection of information [2]. These fundamentals of mental processes between sensory stimulus and the outward display of behavior through researching human cognitive processes are essential from the perspective of information processing theory. The cognitive basis of this theory suggests that various processes, like active processing of information, attention, encoding, storage, and retrieval, are involved in the learning process. Hence, educators utilize strategies like chunking, rehearsal, and elaboration to enhance their students’ cognitive processes [3].

The Merriam-Webster dictionary defines attention as “an instance of focused awareness on a certain amount of the available perceptual information” [4]. The process of attention filters out ineffective information and directs important one to intellectual processes. Moreover, the science of psychology presents solutions to improve focus and complete attention, enhancing the understanding of how students form, store, and recall information [5]. This knowledge of how these mental processes work can assist educators in planning lessons by utilizing appropriate teaching strategies to facilitate students, generate attention and memories, and avoid potential issues with memory consolidation [6]. When planning to improve cognitive functions, pre and post-test settings at the start and end of sessions, pop-up quizzes, use of mnemonics, trigger questions, spaced repetition, and chunking of information in different segments of teaching sessions can be added [7]. Additionally, practicing memorization/repeated recall of working memory in class can effectively increase long-term retention of information in students. Based on schema theory, where new information fits into the existing cognitive structures, educators and facilitators can enable the students to activate the relevant schema and connect the dots between old and new information [3, 8].

It helps to empower and support students in all modes of learning, and a professional teacher must help students develop critical thinking, problem-solving, teamwork, collaboration, and creativity skills [9, 10]. Zone of proximal development, developed by Vygotsky, refers to the difference between what a learner can do independently and what they can achieve with the help of a skilled teacher. While teaching the courses, educators may encounter students who reflect on their learning and alter how they view the world. These students might get in touch with their teachers to reflect on whatever they experience in the real world, connecting with the content taught. This theory supports the significance of scaffolding and social interaction in learning. On the other hand, social cognition theory emphasizes the role of observational learning, self-efficacy, and social interaction. Based on these theories, educators can provide opportunities for students to collaborate and learn from each other. This provides the best possible outcome of successful instruction based on the principles of cognitive psychology [10].

Moreover, misconceptions, ineffective learning strategies, constraints of limited attention and working memory, fear, and mistrust are said to be the main challenges in teaching. Cognitive load theory suggests that instructional design should consider working memory limitations and maintain the cognitive load. To do so, the development and application of cognitive concepts that encourage generalization and application of knowledge should be prioritized [2, 11]. Educators should design learning activities, reading material, and PowerPoint presentations aligned with Mayer’s multimedia principles. The online environment requires a higher level of technical competency on top of cognitive load [10], which can be dealt with by engaging learners using cues and tests and involving them in different activities that increase their attention span [2]. Over and above, constructive feedback about assignments, the implementation of fair policies, the supply of required educational resources, and the availability of educators at the time of guidance can help establish an environment of trust and motivation among students to learn [2].

With a keen interest and a thorough literature review, it was found that there is a dearth of research reporting undergraduate medical and dental students’ perspectives on applying psychological principles in their learning environment. Therefore, it was hypothesized that there is no significant difference in the perspectives of undergraduate medical and dental students regarding the application of psychological principles in their learning environment. This study aimed to assess students’ perceptions regarding applying principles of CP in their teaching, which may help with long-term memory retention and contribute to students’ emotional well-being and satisfaction with teaching practices. The study may highlight the specific attributes of CP that students perceive as beneficial, such as overcoming cognitive and emotional challenges, improving attention, and enhancing knowledge integration. It will also explore the differences in perception between MBBS and BDS students regarding the application of CP principles. The study opens avenues for further research, such as longitudinal studies, to examine the long-term impact of CP principles on student outcomes. It also encourages investigations into the effectiveness of specific CP interventions in different educational contexts. By providing empirical evidence and practical insights, this study contributes to the ongoing dialogue about how to effectively apply cognitive psychology principles to improve medical and dental education.

## Methods

### Study design and setting

A descriptive cross-sectional study was conducted in medical and dental colleges of Karachi involving all years of BDS and MBBS students within a duration of 6 months from May to October 2023 after obtaining approval from the Ethical Review Board of Bahria University Medical & Dental College ((Ethical Review #: FRC/BUHS 37/2022).

### Sampling technique and sample size

Purposive sampling was employed with a sample size of 351 calculated via Open Epi (population size (n) of 4000 and confidence levels of 95% and 5% margin of error). All MBBS and BDS students were included except those who did not go through online classes and did not consent to participate in the study.

### Study instrument

A structured questionnaire was developed by medical educationists and authors of the study (having expertise in the field). The content validity was determined by two medical educationists and a senior medical college professor.

### Validity of the questionnaire

The questionnaire was distributed using a Google Form link to MBBS (n-153) and BDS (n = 130) students. The data used to validate the questionnaire was not included in this study. Validity was ensured by exploratory factor analysis (EFA) through SPSS v26 using the Principal Component Analysis data extraction method. Utilizing the varimax approach, factors with an eigenvalue greater than 1.0 were retained for rotation. For an item to be considered to load on a factor, a criterion level of 0.40 was set. The items with poor loading values (i.e., <0.40) were omitted from further analysis. Sampling adequacy was determined by Kaiser–Meyer–Olkin (:0.889). There were 6 factors loaded on a cumulative eigenvalue of 68.92%. This ensures that the questionnaire aligns with the cognitive psychological principles.

Then, the final questionnaire (link to Google Form) was modified and circulated among the study population via various social media platforms. The questionnaire’s reliability was then confirmed via Cronbach alpha (α: 0.942), indicating a strong reliability level on data from 50 students, which is not included in this study’s results.

The structured questionnaire was divided into two sections. The first section was concerned with demographic details, program, and year of study. The second section included six subsections with 59 items altogether, which included integration of knowledge (containing 10 items), attention during the class (containing 13 items), interaction with the facilitators (containing 06 items), strategies to overcome cognitive challenges (consisting of 07 items), effective learning strategies (consisting of 13 items) and strategies to meet emotional challenges (containing 10 items). All the subsections were assessed using a 5-point Likert scale (0–5: not applicable, strongly disagree, disagree, neutral, agree, strongly agree). In the analysis, the scale was hinged into (not applicable, strongly disagree, strongly agree), and the responses marked neutral on the Likert scale were removed during data analysis. They did not have any positive or negative impact on the final results.

#### Ethical concerns

Each student was informed of the purpose of the research, and a statement regarding their voluntary participation in the study was given at the start of the online questionnaire. It was mentioned that filling out the questionnaire would be considered their consent to participate in the study. Furthermore, all participants were ensured that their confidentiality would be maintained and that the data would be used for research purposes and publication. It was also mentioned that they are free to withdraw from the study at any point in time without any repercussions.

### Statistical analysis

Data was analyzed on SPSS version 21. The categorical variables were represented in frequencies and percentages. The age of the students was represented by mean and standard deviation. The Kolmogorov-Smirnov test was used to check the normality, showing that the data was not normally distributed (p-value < 0.05). Mann-Whitney U test was applied to the attribute scores of both BDS and MBBS students. The attributes were considered significant if the p-value was < 0.05.

## Results

Seven hundred responses were received through an online submission link, and one hundred forty-five responses were excluded based on incomplete responses and repetition. There were 63.4% MBBS and 36.6% BDS students in the study, including 320 (57.65%) females and 235 (42.35%) males. The mean age of participants was 20.55 ± 1.86 years **(Table 1).**

**Table 1:** Descriptive characteristics of study participants (N = 555).

Variable	Mean±SD	
Age	20.55±1.86	
	**Frequency (%)**	
Gender	**Male**	**Female**
	235 (42.4)	320 (57.6)
Program	**MBBS**	**BDS**
	352 (63.4)	203 (36.6)

**Fig 1:**
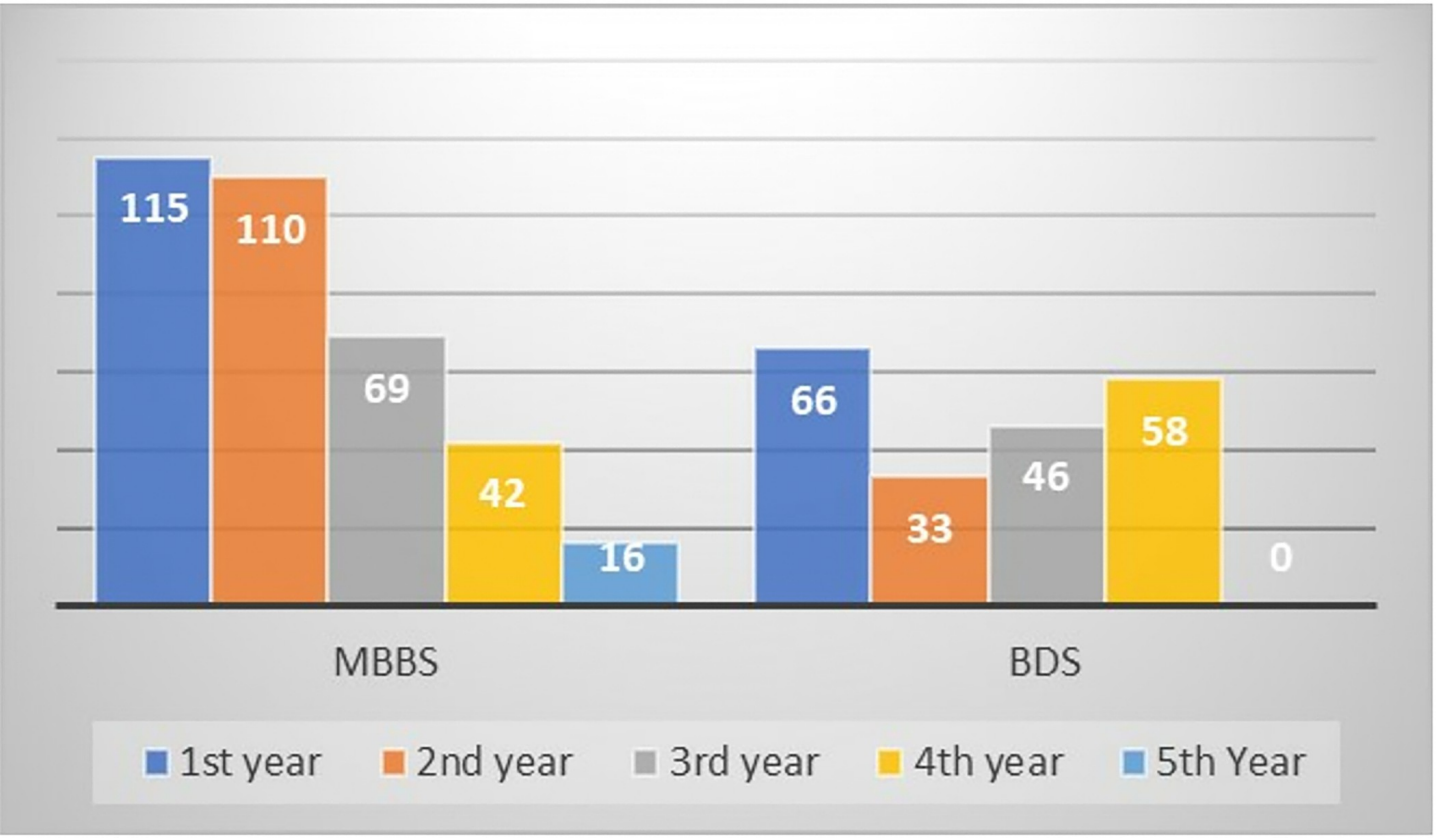
Study year-wise distribution of MBBS and BDS students.

The representation of basic sciences years was higher in MBBS. In contrast, in BDS, the first and final years participated more than the mid-years (Fig 1).

Most MBBS and BDS students were confident regarding integrating their knowledge and facilitator competence to teach in person. The majority were satisfied with the teaching and formative assessment when examining the attributes of attention in the class. However, most MBBS and BDS students agreed that they did not receive breaks between their teaching sessions to maintain attention. The results also indicated that BDS students were lacking in course content presentation, sequencing, and coverage compared to MBBS students. The third attribute, the interaction of the facilitators with students showed a lack of communication with the use of technology (emails, online discussion forums, and chat boxes) and online learning platforms by the facilitators. BDS students here also responded that they had less facilitator feedback than MBBS students. Moreover, in the fourth attribute, which covers strategies used to meet cognitive challenges, it was observed that students did not receive reading material like handouts before class; they were not sure that they had the chance to review pre-assessment results and take part in teams to complete projects. Most students also disagreed regarding getting feedback within the larger class. In this attribute, BDS students reported having fewer opportunities to meet these challenges than MBBS students. The fifth attribute showed strategies to overcome ineffective learning; comparing MBBS to BDS students, both disagreed with having the flavor of online teaching, frequent quizzes, and needing to learn about mnemonics, whereas BDS students seem to have less exposure than MBBS students. Moreover, the same was reflected in the sixth attribute responses from students reporting that the facilitators were less available and supportive for BDS students as compared to MBBS students and were less tech-savvy when dealing with online platforms if they used it somehow (Table 2). This table represents responses lying in not applicable, strongly agree (agree merged), and strongly disagree (disagree merged) areas; however, neutral responses were not mentioned but can be calculated. The reason for not mentioning neutral responses and their interpretation is to have a better understanding of trends in opinions, as these responses can reflect indecisiveness rather than a meaningful position. By focusing on non-neutral responses, the analysis highlights stronger patterns, enabling more actionable insights while minimizing ambiguity.

**Table 2:** Comparison of self-perceived application of psychology principles of MBBS and BDS students.

Statements	MBBS (352)			BDS (203)		
**1. Integration of knowledge**	*NA	*SA	*SD	**NA**	**SA**	**SD**
Complemented mindset growth	24(6.8)	208 (59)	31(8.8)	15 (7.4)	106 (52.2)	15 (7.4)
Developed content understanding	15(4.3)	268(76.1)	27(7.7)	7(3.4)	133 (65.5)	20(9.9)
Encouraged making notes	13(3.7)	215(61.1)	58(16.5)	9(4.4)	118(58.1)	31(15.3)
Enhanced engagement	13(3.7)	226(64.2)	42(11.9)	8(3.9)	106(52.2)	29(14.3)
Improved learning and confidence	7(2.0)	252(71.6)	36(10.2)	3(1.5)	138(68.0)	21(10.3)
Promoted positive belief	8(2.3)	264(75)	29(8.2)	5(2.5)	144(70.9)	20(9.9)
Connected with prior knowledge	9(2.6)	239(67.9)	49(13.9)	5(2.5)	118(58.1)	26(12.8)
Connected content in other situations	3(0.9)	249(70.7)	47(13.4)	2(1.0)	135(66.5)	19(9.4)
Helped to reach to the solution	7(2.0)	244(69.3)	38(10.8)	4(2.0)	117(57.6)	23(11.3)
Critically analyze information	6(1.7)	238(67.6)	42(11.9)	3(1.5)	115(56.7)	24(11.8)
**2. Attention during the class**	NA	SA	SD	NA	SA	SD
Grasped attention	9(2.6)	190 (53.9)	69(19.6)	5(2.5)	85 (41.9)	37(18.2)
Content presented in logical order	4(1.1)	228(64.8)	55(15.6)	3(1.5)	117(57.6)	25(12.3)
Connection to real-life practices	5(1.4)	234(66.5)	52(14.8)	3(1.5)	151(74.4)	13(6.4)
Legible content presentation	7(2.0)	249(70.7)	55(15.6)	3(1.5)	144(70.9)	16(7.9)
Content related pictures, etc.	7(2.0)	270(76.7)	27(7.7)	3(1.5)	155(76.4)	16(7.9)
Important concepts highlighted	6(1.7)	263(74.7)	30(8.5)	2(1.0)	136(67.0)	20(9.9)
Questioning improved attention	9(2.6)	262(74.4)	28(8.0)	4(2.0)	137(67.5)	15(7.4)
Appreciation motivated to learn	8(2.3)	266(75.6)	28(8.0)	3(1.5)	147(72.4)	21(10.3)
Short breaks during the session	28(8.0)	147(41.8)	115(32.7)	13(6.4)	80(39.4)	66(32.5)
Pop-up quizzes	8(2.3)	252(71.6)	45(12.8)	5(2.5)	106(52.2)	32(15.8)
Discussions enhanced knowledge retention	6(1.7)	271(77.0)	37(10.5)	3(1.5)	128(63.1)	20(9.9)
Quizzes revision improved retention	13(3.7)	246(69.9)	49(13.9)	6(3.0)	112(55.2)	28(13.8)
Self-assessment questions provided	13(3.7)	224(63.6)	66(18.8)	8(3.9)	103(50.7)	38(18.7)
**3. Interaction with the facilitators providing motivation**	NA	SA	SD	NA	SA	SD
Verbally in the class	6(1.7)	286(81.3)	20(5.7)	4(2.0)	163(80.3)	9(4.4)
E-mails	27(7.7)	99(28.1)	161(45.7)	24(11.8)	38(18.7)	87(42.9)
Discussion forums	20(5.7)	189(53.7)	77(21.9)	17(8.4)	83(40.9)	51(25.1)
Chatbox (synchronous sessions)	28(8.0)	152(43.2)	110(31.3)	23(11.3)	47(23.2)	69(34.0)
Regular feedback	16(4.5)	216(61.4))	52(14.8)	9(4.4)	113(55.7)	20(9.9)
Appreciation on performance	9(2.6)	279(79.3)	32(9.1)	5(2.5)	150(73.9)	14(6.9)
**4. Strategies used to meet cognitive challenges**	NA	SA	SD	NA	SA	SD
Adherence to the course outline	2(.6)	235(66.8)	55(15.6)	0(0.0)	133(65.5)	40(19.7)
Reading materials before the class	6(1.7)	171(48.6)	127(36.1)	64(31.5)	47(23.2)	80(39.4)
Review pre-assessment results	2(0.6)	173(49.1)	140(39.8)	39(19.2)	46(22.7)	87(42.9)
Teamwork to complete projects	1(0.3)	199(56.5)	101(28.7)	28(13.8)	59(29.1)	83(40.9)
Read first and then discuss	2(0.6)	179(50.9)	109(31.0)	3(1.5)	123(60.6)	28(13.8)
Session corrected the concepts	5(1.4)	250(71.0)	29(8.2)	3(1.5)	94(46.3)	77(37.9)
Feedback on the task	1(0.3)	174(49.4)	131(37.2)	17(8.4)	41(20.2)	106(52.2)
**5. Strategies to overcome ineffective Learning:**	NA	SA	SD	NA	SA	SD
Quizzes within the class	4(1.1)	243(69.0)	54(15.3)	1(.5)	110(54.2)	39(19.2)
Quizzes provided online	22(6.3)	118(33.5)	135(38.4)	11(5.4)	58(28.6)	95(46.8)
Prerecorded lecture videos online	25(7.1)	87(24.7)	185(52.6)	17(8.4)	33(16.3)	122(60.1)
Motivated to enhance learning	4(1.1)	250(71.0)	31(8.8)	1(.5)	121(59.6)	16(7.9)
Prior knowledge of the content	4(1.1)	181(51.4)	56(15.9)	3(1.5)	89(43.8)	32(15.8)
Connection between knowledge	1(.3)	239(67.9)	33(9.4)	8(3.9)	123(60.6)	72(35.5)
Multiple modalities used	7(2.0)	250(71.0)	35(9.9)	2(1.0)	137(67.5)	18(8.9)
Visual presentations in the class	3(0.9)	231(65.6)	36(10.2)	2(1.0)	131(64.5)	21(10.3)
Concepts constructed on prior knowledge	4(1.1)	255(72.4)	21(6.0)	0(0)	133(65.5)	15(7.4)
Mnemonics provided	6(1.7)	197(56.0)	75(21.3)	3(1.5)	85(41.9)	47(23.2)
Focus on the organization of topic	4(1.1)	220(62.5)	53(15.1)	0(0)	115(56.7)	31(15.3)
Relate new information	5(1.4)	245(69.6)	36(10.2)	1(0.5)	140(69.0)	15(7.4)
Rehearse the knowledge	1(0.3)	261(74.1)	39(11.1)	2(1.0)	127(62.6)	11(5.4)
**6. Strategies used to meet emotional challenges:**	NA	SA	SD	NA	SA	SD
Knowledgeable and competent	9(2.6)	294(83.5)	15(4.3)	3(1.5)	162(79.8)	6(3.0)
Approachable during office hours	3(0.9)	296(84.1)	18(5.1)	0(0)	146(71.9)	16(7.9)
Truthful and respectful	6(1.7)	303(86.1)	17(4.8)	1(0.5)	158(77.8)	8(3.9)
Fair to policies and deadlines	5(1.4)	301(85.5)	17(4.8)	1(0.5)	157(77.3)	7(3.4)
Unbiased in providing feedback	8(2.3)	262(74.4)	26(7.4)	0(0)	128(63.1)	15(7.4)
Discuss and share experiences	7(2.0)	270(76.7)	24(6.8)	1(.5)	141(69.5)	17(8.4)
Tech-savvy	20(5.7)	185(52.6)	71(20.2)	12(5.9)	26(12.8)	42(20.7)
Taught relevant topics	8(2.3)	285(81.0)	26(7.4)	2(1.0)	166(81.8)	11(5.4)
Adequate learning resources	5(1.4)	272(77.3)	37(10.5)	1(0.5)	143(70.4)	21(10.3)
Informal discussion forum	8(2.3)	227(64.5)	68(19.3)	9(4.4)	102(50.2)	44(21.7)

NA = not applicable, SA = strongly agree, SD = strongly disagree, SGD = small group discussion

Table 3 tested the present study hypothesis that there is no significant difference between BDS and MBBS students’ perspectives regarding applying the psychological principles in their learning environment. However, this null hypothesis was rejected, and significant differences were found in all attributes except one (p < 0.05). After applying Bonferroni correction, the integration of knowledge and attention during the class no longer stayed significant. However, the other three attributes suggested that MBBS students are better at overcoming routine and emotional challenges and also significantly better at recognizing and overcoming ineffective learning strategies than BDS students. In an attribute "interaction with the facilitators," no significant difference was observed between MBBS and BDS students (p = 0.057).

**Table 3:** Comparison between scores of attributes regarding the application of psychology principles in BDS and MBBS students.

Attributes (Total items) *(adjusted alpha*: *0*.*00837)*	BDS	MBBS	p-value	U	Z	Median (IQR)
	Mean Rank					
1. Integration of knowledge (10)	256.15	290.60	0.015*	31293.00	-2.44	37(8)
2. Attention during the class (13)	256.62	290.33	0.017*	31388.00	-2.38	49(13)
3. Interaction with the facilitators (6)	260.97	287.82	0.057	32271.00	-1.90	20(6)
4. Strategies to overcome cognitive challenges (7)	188.12	329.84	0.001*	17482.00	-10.05	22(7)
5. Overcome in effective learning strategies (13)	228.35	306.63	0.001*	25648.50	-5.54	46(12)
6. Strategies meet emotional challenges (10)	241.26	299.19	0.001*	28270.50	-4.110	39(8)
Total scores (59) ***(r = 0*.*798)***	109.46	375.20	0.001*	1514.50	-18.80	343(193)

*p<0.05 was considered significant

The total scores’ effect size indicates a large effect (r = 0.798), which represents both statistically significant and meaningful in practical terms.

## Discussion

All educational practices are supported by educational philosophy and learning theory because they offer the conceptual frameworks for describing how a person acquires the knowledge, skills, and attitudes necessary to achieve changes in behavior, performance, or potential [12].

The current study focused on assessing students’ perceptions regarding applying principles of CP in their teaching, which may help with long-term memory retention and contribute to students’ emotional well-being and satisfaction with teaching practices. The present study findings indicated that MBBS students’ perceptions were significantly better than those of BDS students in five out of six attributes of CP (overcoming cognitive and emotional challenges, recognizing and overcoming ineffective learning strategies, paying attention during class, and integrating knowledge).

The present results can be explained by the fact that MBBS and BDS programs have different curricula, course durations, and numbers of students. These factors may significantly influence students’ perceptions. Another reason could be that the MBBS students’ institution regularly supports students facing emotional and cognitive challenges. It is also possible that the MBBS curriculum is lengthy and comprehensive, so students are mentally prepared to overcome cognitive and emotional difficulties, adopt effective learning strategies, pay attention and, participate in class discussions, and integrate previous knowledge with new knowledge.

It was observed in this study that sharing prerecorded lecture videos and working in teams did not help our students prepare the content before class, access the lectures at their convenience, and review the key concepts, which contradicts the literature [13, 14]. However, they mentioned that the facilitators helped them cater to cognitive challenges within the class. This could be because the students come from different educational and sociocultural backgrounds, each with a different learning style, thus hindering the teamwork required to prepare before class.

Current research findings showed that encouraging students to take notes during class and assisting them in doing so improved their ability to integrate their knowledge and create a solid comprehension of the subject. Students integrated their knowledge by building upon prior knowledge, connecting dots, critically appraising the information, and putting together the parts of a larger puzzle. They enhance their engagement, develop a strong self-belief, and boost their overall confidence. The provision of mnemonics as instructional material by the facilitators helped in the retention of information [15] that is not provided to our students, most likely due to the lack of creativity, training, or unawareness of the facilitator on the cognitive impact of mnemonics on learning. Therefore, this highlights the dire need for training workshops with clear objectives of creating awareness and training facilitators in CP principles and how to incorporate them into their teaching methodologies. Routh et al. in their study, developed a tool to assess the preparedness of veterinary students by application of cognitivism and its various stages of learning [16].

Academic engagement of students has been identified in the literature as a key feature driving the students’ motivation and success [16]. Singh et al., in their study, identified several active teaching strategies like flipped classrooms and team-based learning to enhance student motivation and engagement [17]. In a study by Talan et al., flipped classroom and blended learning models enhanced student engagement, increasing their overall class participation and attention level [18]. These findings concord with the results of the present study, whereby students of both MBBS and BDS stated that they were encouraged to participate in class, which may have increased their engagement. However, they did not read the material before the class. This evidence in itself points towards the risk of social desirability bias. However, the chances of this were minimized using validation, anonymizing the data and the researcher identities, and also by adopting a Google forms form for data collection and neutral wording of questions.

The foundation for designing effective multimedia presentations lies in the cognitive theory of multimedia learning, which implies that individuals learn from pictures rather than words [19]. Literature suggested using visual representation, pictures, diagrams, videos, and animations and foregoing the use of bullet points to increase audience attention span [19]. Concerning this evidence in the literature, the current study results also reveal increased attention of MBBS and BDS students during lectures as their teachers follow multimedia principles. Moreover, in this study, students perceived that the short breaks were little in practice; however, the fact that facilitators provided pop-up quizzes kept them engaged. These findings are in corroboration with several other authors. Paulus et al. found that standing breaks in lectures increased university students’ concentration, receptiveness, and retentiveness, and the students highly accepted these breaks [20]. Literature suggests that the “*testing effect*” improves learning and enhances retention [21]. Evidence points towards the significance of formative assessments as they help the learner develop a deeper insight into the topic, identify the gap between their performance and the standard required, and help develop approaches that can help bridge the gap [22].

Feedback is *"communication of information relating to a person’s performance in a particular activity that is intended to help their future presentation in the same or linked activity*.*"* Just like the notion that assessment drives learning, regular feedback and constant motivation by the teacher help the students develop confidence in their learning and enhance the development of long-term memory [6, 22]. Participants of the current study reported getting less instant feedback during the larger group teaching; however, regular and unbiased feedback provided by facilitators in other sessions motivated them to learn. Ahmady et al. identified in their systematic review a moderately significant relation between motivation and academic performance and achievement of students [23]. Studies have found that providing feedback on reasoning and problem-solving during preclinical to early clinical training can improve student performance and clinical practices [24, 25].

Some facilitators in the present study were not well versed in technology like Zoom and voiceover, which caused disruptions. Interactive teaching technology in higher education effectively improves students’ preparedness and learning outcomes [26, 27]. The interaction principle can increase medical students’ teaching and learning, regardless of the mode of delivery, address the potential limitations of other forms, and contribute significantly to better mental health [27]. However, this limitation of some facilitators led to a lack of student engagement, loss of real-time learning experiences, and lack of collaboration between the two stakeholders, eventually leading to frustration and distrust. Moreover, it can also lead to impaired development of skills in terms of technology usage. However, this challenge can be resolved through professional training and workshops and investing in the infrastructure. These can help develop an innovative environment where facilitators can discuss and help each other overcome these challenges, fostering a culture of improvement, enhancement, and development. This shortcoming can also be overcome by making technology a formal part of the curriculum with clearly designed and measurable objectives.

Theories underpinning interactive lectures, cognitivism, and constructivism form cornerstones in changing the old traditional lecture forms to a more student-centered approach [28]. Our study results also reflect increased motivation of students when the facilitator was interactive, gave them feedback, and appreciated their performance in class compared to connecting through emails, discussion forums, or employing technology.

### Implications and recommendations

The current study indicates that CP application principles are relevant for undergraduate medical and dental students. However, there is a need to arrange regular workshops for the faculty on interacting with students using discussion forums, chat boxes, and other tools, as such interaction with facilitators motivates students. Moreover, training workshops should be arranged for faculty members to hone their skills in technology, which can contribute to better application of the principles of CP. Additionally, faculty should be trained in strategies to address ineffective learning by preparing and uploading repeated quizzes online using different apps or providing them as handouts for practice, as well as recording and uploading interactive lecture videos online. Small group sessions should also be arranged to train students in strategies to overcome ineffective learning by studying the provided material before scheduled sessions, thereby maximizing their understanding and learning during the session. There is a dire need to guide students in strategies to meet cognitive challenges so they can handle the tremendous information load in medicine and dentistry. Additionally, students must learn how to participate and work in teams, as teamwork will significantly benefit them in their real-life work.

Lastly, if institutes wish to apply the principles of CP, they should adopt blended learning approaches. Institutes should have a comprehensive learning management system to maximize student and faculty benefits. Moreover, to gain the maximum benefits from CP principles, there is a need for curricular changes and the inclusion of case-based learning, team-based learning, and problem-solving activities that align well with CP principles. In these techniques, students are encouraged to retrieve and integrate knowledge and apply critical thinking skills to solve cases. These techniques also maximize student interaction with content and improve engagement and cognitive skills. Future research might consider a longitudinal design to capture changes in student perceptions over time, providing a more comprehensive view of how cognitive psychology principles impact learning in the long term.

### Limitations

The students’ responses were collected on a self-reported questionnaire; hence, the possibility of recall bias at the time of administration of the questionnaire cannot be ignored. The results may be subject to variability based on the different learning styles of students and the use of different teaching modalities for MBBS and BDS students. Additionally, this study is quantitative, and it would not be possible to gain deeper insights about the topic from participants without including qualitative methods, such as focus group discussions, open-ended questions, interviews, and so on. Furthermore, it is worth noting that there were more females than males in the present study, which may influence the results due to this gender disparity. As the study relies on a self-reported questionnaire, only the students’ perceptions are noted, and actual improvements in critical thinking, motivation, and memory retention cannot be measured directly. A purposive sampling technique was used that may introduce selection bias. It is important to consider that this study collected data at a single point in time, thus preventing the examination of the long-term effects of applying CP principles in teaching and the potential changes in students’ perspectives over time. Moreover, this being a cross-sectional study cannot relate to the longitudinal impact and sociocultural difference that can shape students’ perceptions of CP principles. However, the study emphasizes that to optimize learning outcomes, institutions should consider adopting blended learning strategies, curricular innovations, and active learning methodologies (such as case-based, team-based, and problem-solving approaches) aligned with CP principles.

## Conclusion

The present study findings indicate that MBBS students’ perceptions were significantly better than those of BDS students in five out of six attributes of CP. Moreover, applying CP principles made the sessions more interactive, augmenting student engagement, attention, and memory. Formative assessment and constructive feedback promoted critical thinking, motivation, and long-term retention among medical students. Proper use of technology, provision of handouts or materials, and a chance to review pre-assessment results and participate in teams to complete projects were a few points that need to be addressed. Future longitudinal research could provide deeper insights into the long-term impact of CP principles on student learning and perception.

## Supporting information

S1 File MBBS dataset. (CSV)

S2 File BDS dataset. (CSV)
